# Robot-Assisted Carotid Artery Stenting: A Safety and Feasibility Study

**DOI:** 10.1007/s00270-020-02759-0

**Published:** 2021-01-14

**Authors:** Ben Jones, Celia Riga, Colin Bicknell, Mohamad Hamady

**Affiliations:** 1 Department of Interventional Radiology, Imperial Health and Academic NHS Trust, London, W2 1NY UK; 2 Department of Surgery and Cancer, Imperial College, London, W2 1NY UK; 3 Imperial Vascular Unit, St Mary’s and Charing Cross Hospitals, Imperial Healthcare NHS Trust, London, UK

**Keywords:** Carotid artery stent, CAS, Robot, Endovascular robotics

## Abstract

**Purpose:**

Endovascular robotics is an emerging technology within the developing field of medical robotics. This was a prospective evaluation to assess safety and feasibility of robotic-assisted carotid artery stenting.

**Materials and Methods:**

Consecutive cases of carotid artery stenting cases performed over period of 24 months, from May 2015 to October 2016, using the Magellan Robotic System (Hansen, Mountain View, CA) were included. All cases utilised the robotic system to navigate the arch, obtain a stable position in the common carotid artery, followed by manual manipulation of Embolic Protection Devices and self-expandable stents through the robotic catheter. Patients demographics, clinical indications, anatomical features, technical and clinical success, complication rate and hospital stay were prospectively recorded.

**Results:**

Thirteen patients, 10 males (78.5%), with an average age of 68.7 years were treated. Mean follow up time was 30 months. Ten patients (91%) were symptomatic at presentation. Anatomical indications for endovascular stent insertion were previous open surgery to the neck ± radiotherapy (87.5%) and hostile anatomy for open surgery (12.5%). Technical success was 100% and the robotic system demonstrates enhanced stability during arch and lesion crossing. There were no neurological complications post-operatively. Average hospital stay was 3 days (range 2–6 days) and a change in serum creatinine of −7.8 μmol/L. There was no documented case of in stent restenosis, new or worsening neurology during follow-up.

**Conclusion:**

These results illustrate safety and feasibility of robotic endovascular revascularisation for carotid disease and demonstrates potential to enhance peri-procedural safety through improved control and stability.

## Introduction

Carotid artery stent (CAS) has been considered an alternative to carotid endarterectomy (CEA) in the presence of “hostile” anatomical and/or medical factors precluding open surgery [1, 2].

Aortic arch type and configuration, vessel tortuosity, atheroma burden (in both the target and access vessels) as well as length of stenosis significantly affect guide wire/catheter manipulation and appear to be the major factor contributing to inherent neurologic risk associated with the procedure [3, 4].

Research from our institution has demonstrated improved catheter manoeuvrability, accuracy and stability as well as reduction of access to target path, reduction of catheter-wall contact and subsequent reduction of high intensity signals recorded on transcranial doppler for endovascular robotic catheter technology [5–9].

This prospective case series aims to evaluate the safety and feasibility of endovascular robot-assisted carotid artery stent using Magellan system.

## Materials and Methods

Between May 2015 and October 2016, all patients referred to our unit for CAS were recruited. Demographics, anatomical and clinical data were collected using electronic information system (Table 1). Using CT angiography, anatomical assessment was performed and included; aortic arch type and angle, severity of arch atheroma, carotid artery tortuosity index (TI), degree of carotid stenosis and lesion length. Data were analysed by a qualified vascular Interventional Radiologist (MH), using semi-automated reconstruction software (Endosize; Therenva, Rennes, France).

**Table 1:** Patients demographics

Demographics	%
Age	45–85 (61)
Sex	12 M
Hypercholesterolemia	28%
Hypertension	42%
Coronary artery disease	42%
Diabetes	21%
Smoking	42%
Prior CABG	14%

**Fig. 1:**
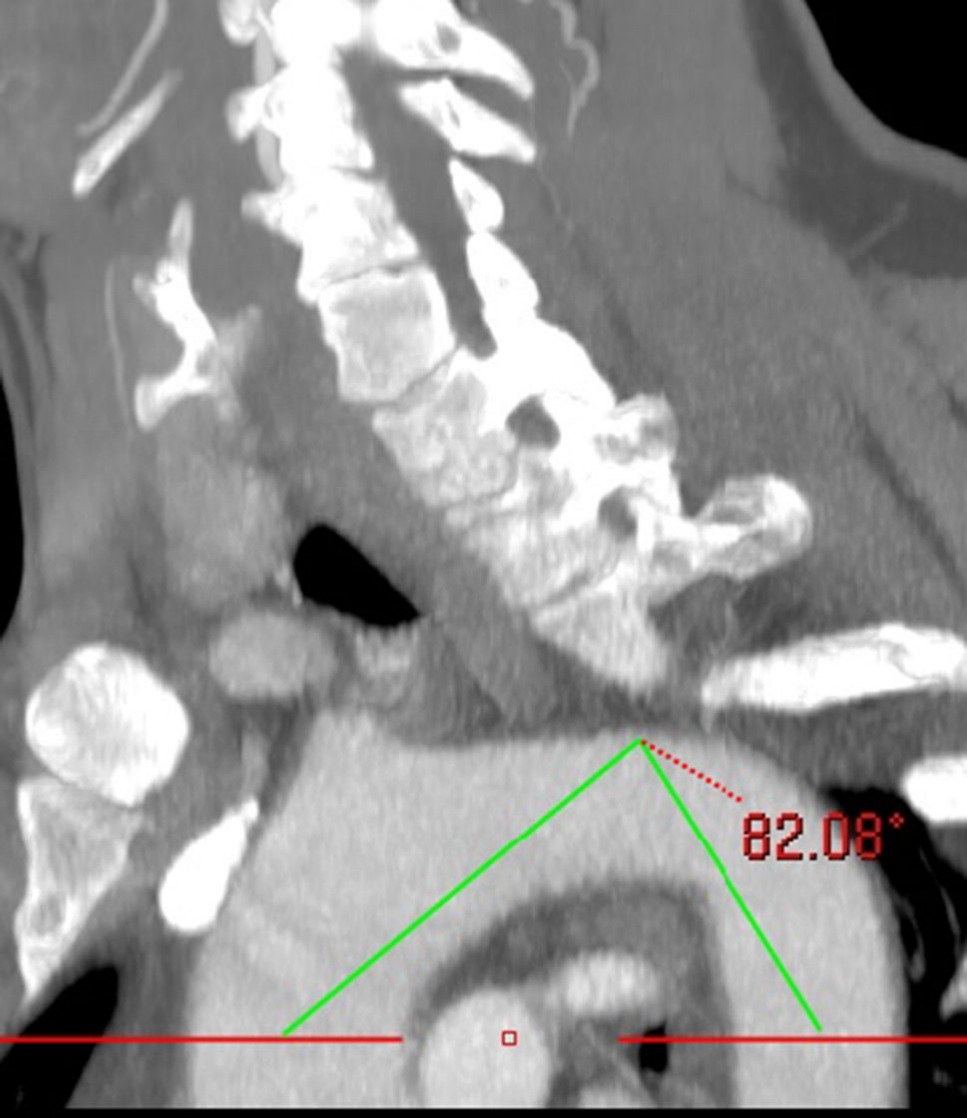
Maximum intensity projection in left anterior oblique position, showing the method of measuring aortic arch angle. A horizontal line is placed at the highest point of the pulmonary trunk and the angle measured between middle point of the ascending aorta, descending aorta and the highest point of the arch

**Fig. 2:**
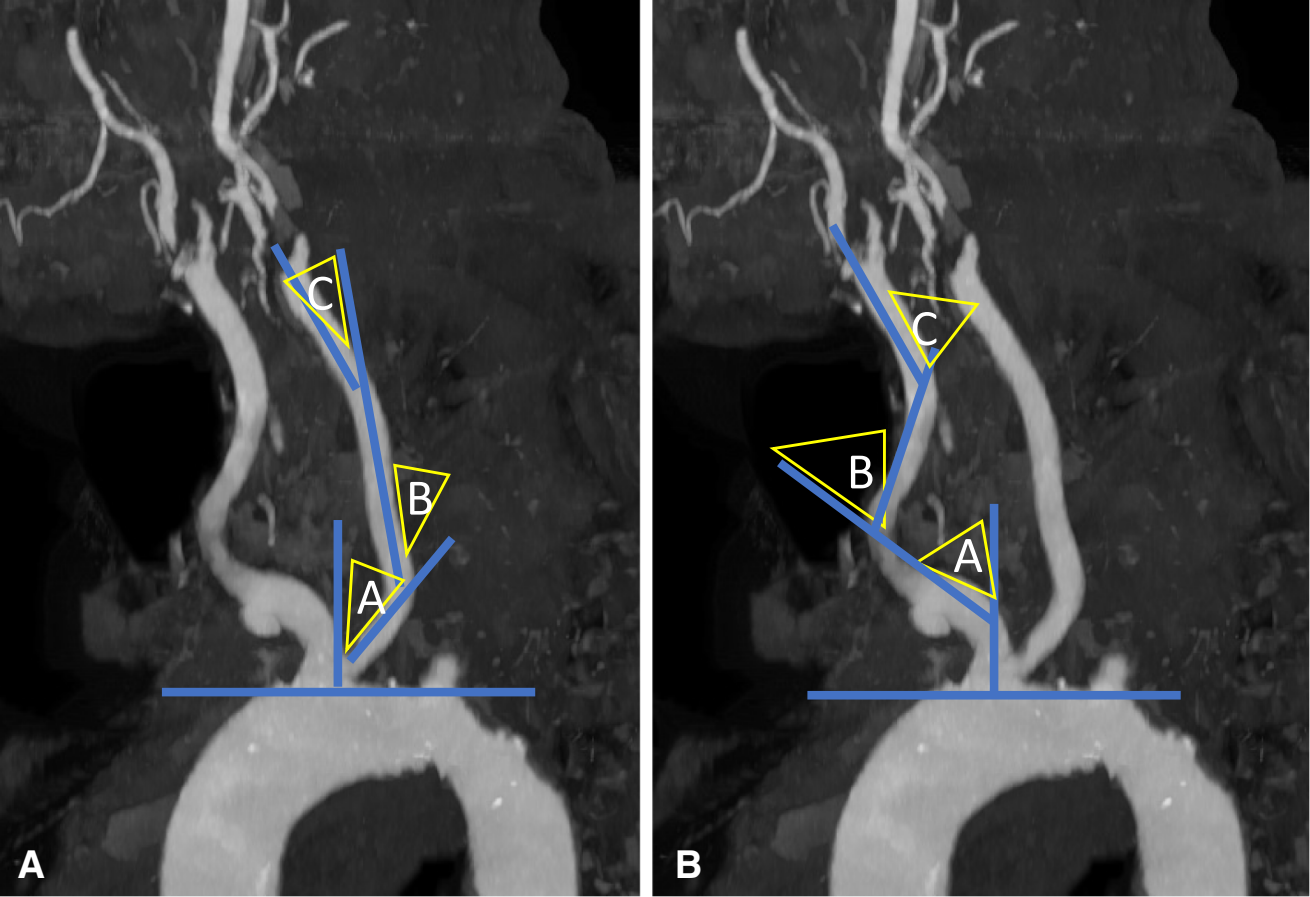
CT angiography with maximum intensity projection in left anterior oblique view. Proximal tortuosity index (TI), **A** right and **B** left, is the mean of A + B + C. **C** Intra-arterial digital subtraction angiography of the right carotid artery. Distal tortuosity index (TI) is the sum of A + B + C angles

Aortic arch type was classified according to the distance of great vessel origin from the horizontal line of the arch [10]. Arch angle was measured with the horizontal line placed at the highest point of the pulmonary trunk and the angle measured between middle point of the ascending and descending aorta and the highest point of the arch [11] The carotid tortuosity index (TI) was calculated as the sum of divergent angles obtained from straight line across the origin of innominate or left common carotid artery against the tangent of the superior surface of the aortic arch [12] (Figs. 1, 2).

The severity of arch atheroma was assessed using 5 point-scoring system [13, 14]. The degree of lesion stenosis was assessed according to NASCET criteria.

Hostile anatomy was considered in the presence of one or more of the following; arch type III, arch angle <90°, proximal TI > 150, distal TI > 150. The common and internal carotid artery tortuosity index was also applied using the semi-automated method [12]. Technical success was defined as insertion, vessel cannulation, lesion crossing and retrieval of the robotic catheter system together with successful stent angioplasty of the carotid stenosis and retrieval of the filter protection system. Clinical success was defined as absence of major adverse event post lesion revascularisation of carotid stenosis. Major adverse events were defined as stroke, myocardial infarction or death. Consent form for utilisation of endovascular robot was obtained from all patients.

### The Robotic System

The Magellan Robotic System (Hansen Medical, Mountain View, California, USA) is an electro-mechanically based “master–slave” operator system, which facilitates endovascular navigation via a remotely steerable, multi-directional guide catheter using a robotic arm. The technical specifications have been described in detail in the existing literature [5, 6, 8].

### Procedure and Follow Up

**Fig. 3:**
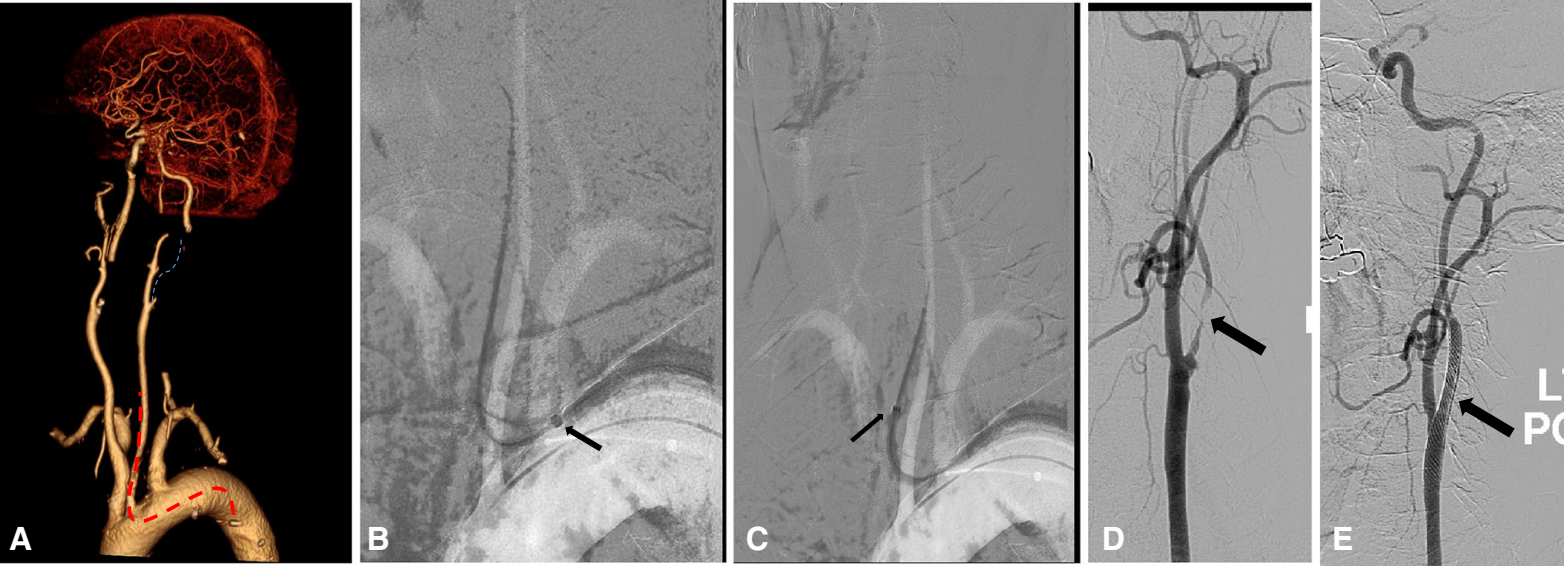
Symptomatic and relatively long left internal carotid artery stenosis. **A** Volume rendering image of CT angiography showing type III arch, long left internal carotid artery severe stenosis (dotted blue line). The red dotted line shows the expected path of the robotic catheter. **B**–**C** Roadmap captures showing the progress path of the robotic catheter (short arrow). **D**–**E** Angiography images showing very severe and long internal carotid artery stenosis before and after carotid stent (arrow)

**Fig. 4:**
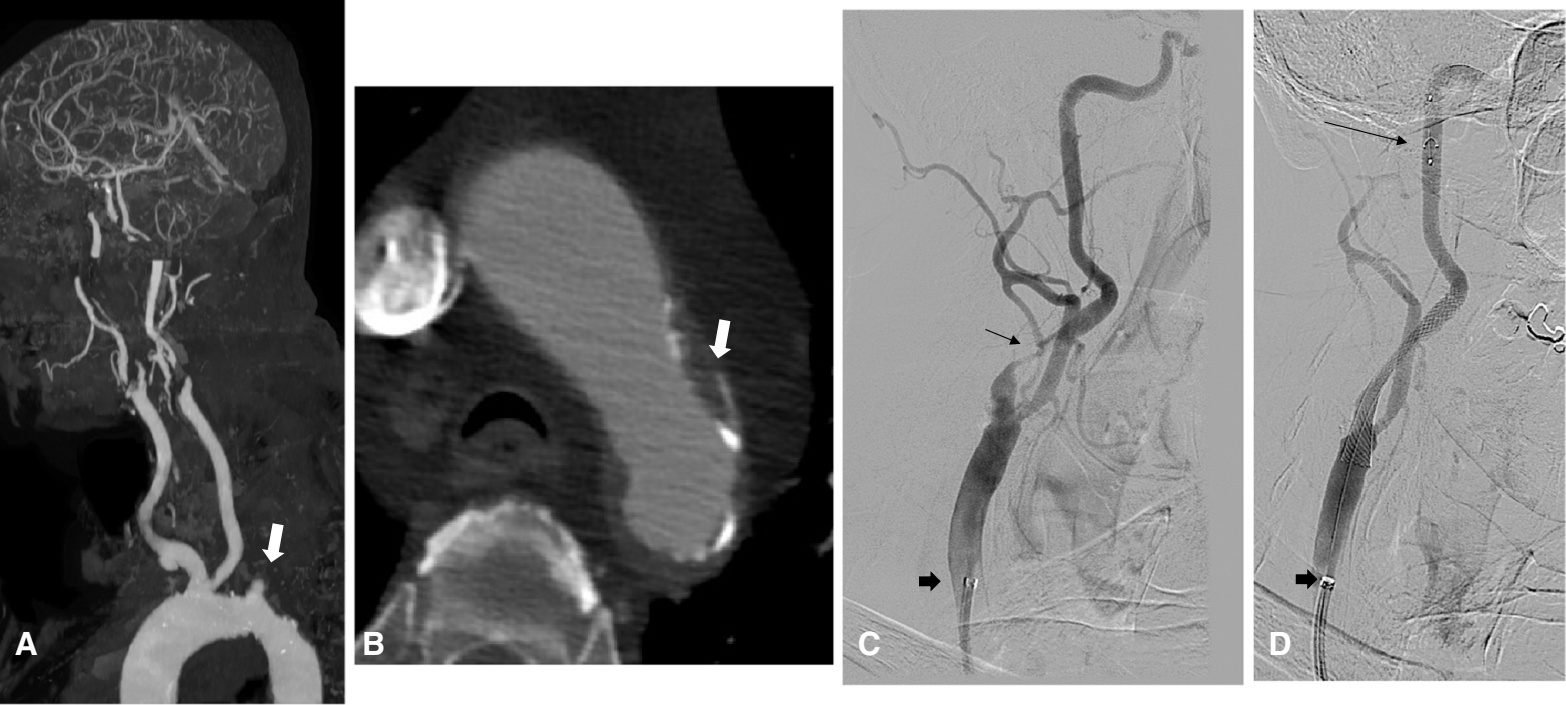
Patient with symptomatic sever bilateral internal carotid artery (ICA) stenoses. **A** Maximum intensity projection of CT angiography showing bovine arch, tortuous innominate artery. **B** Axial image of CT angiography at the level of aortic arch showing grade 4 atheroma (white arrow) just proximal to the bovine origin. **C** Angiography image showing tight ICA stenosis (thin arrow) and robotic sheath in the common carotid artery (thick arrow). **D** Angiography image post CAS showing filter distal protection device (thin long arrow) and stent across the ICA

All procedures were performed in standard angiosuite compatible with the robotic platform. Under local anaesthesia and ultrasound guidance, the robotic catheter was introduced via a 9F access sheath. Navigation and cannulation of the aortic arch and common carotid arteries were performed using the robotic co-axial catheters over 0.35″ hydrophilic wire. The target lesion was crossed manually with an 0014″ wire-based filter system (SpiderFX™, Medtronic). Once the lesion was crossed, filter deployment and stent angioplasty were advanced and delivered through the robotic sheath which acted as a steerable yet stable platform (Figs. 3, 4). Due to the stability of the robotic system, wire placement in the external carotid artery was not necessitated. The robotic sheath could be further adjusted through small, controlled individual movements to further enhance conformability of conventional endovascular tools passing through its lumen whilst minimising contact with the vessel wall. The lesion was predilated with 3 mm balloon and stented appropriately (Carotid WALLSTENT™ Monorail™ Endoprosthesis Boston Scientific Corp. MA). Post stent balloon dilatation was carried out. Completion angiography through the robotic sheath was performed to assess post procedure results. Technical success was recorded when both stent position and revascularization appearances were satisfactory on post treatment angiography. Angio-seal (Terumo, Europe NV) closure device was used to achieve haemostasis. Following discharge, patients were commenced on a dual antiplatelet therapy (daily dose aspirin 75 mg and clopidogrel 75 mg) along with a statin prescribed as their standard peri-operative “best medical therapy” regimen. Clinical success was recorded where a patient did not report any new/worsening neurology and there was no evidence of significant in-stent restenosis during follow-up.

### Statistical Analysis

Statistical analysis was performed using SPSS version 26. Categorical data were presented as percentage and numbers and continuous data as median and interquartile range of mean and standard deviation.

## Results

Prospective data collection was obtained from thirteen consecutive patients who underwent a robot-assisted CAS procedure. Patients demographic were summarised in Table 1. The majority of cases, 77% (10/13), were symptomatic at presentation. Borderline (60%) and sever stenosis were noted in 8 and 92% of patients, respectively.

A grade 1 arch was present in 4 (31%), grade 2 in 1 (8%), grade 3 in 8 (61%). Arch angle was <90° in 77% of patients. Proximal TI > 150 was noted in 46% and distal TI > 150 in 23%. Arch atheroma grade 2 was noted in 46%, grade 3 in 31% and grade 4 in 23% (Table 2).

**Table 2:** Summary of anatomical features (aortic arch type, arch angle, atheroma grade, vessel stenosis, tortuosity index (IT), plague type and lesion length) as well as stent length and procedure time

	*N* = 14
Arch type	
I II III Bovine	36% (5) 0% (0) 57% (8) 7% (1)
Mean arch angle (degrees, SD)	76.64 (15.08)
Atheroma grade	
2 3 4	43% (6) 36% (5) 21% (3)
Median ostial stenosis (range)	0 (0–50)
Mean TI proximal (SD)	119.35 (55.89)
Mean TI distal (SD)	112.08 (49.55)
Median ICA stenosis (%, IQR)	90 (15)
Plaque type	
Noncalcified Heterogenous	64% (9) 29% (4)
Side	
Left Right	50% (7) 50% (7)
Median stent length (IQR)	30 (1.25)
Mean lesion Length (SD)	21.46 (8.90)
Median procedure time (mins, IQR)	75 (15)

Technical success was achieved in all of patients (100%). Lesion crossing was always performed under robotic control and was successful in all cases in under 2 min. Clinical success was achieved in all patients. There were no reported complications during post procedural inpatient stay period apart from one access complication due to failure of the angioseal closure device in an anticoagulated patient which required surgical exploration and repair.

The average screening time was 12.4 min (range 10–21 min) and average procedure time was 74.6 min (range 70–110 min). The radiation dose was estimated as median Air Kerma Air Product of 63 Gy.cm^2^. The average robotic-set-up times was just under 5 min. There was no new or worsening neurology or in stent restenosis, achieving 100% clinical success rate.

## Discussion

Several factors have been considered as potential underlying cause of technical failure and increased peri-procedure risk of stroke during CAS. Arch type, arch angulation, tortuosity, plaque morphology and lesion length are thought to be critical in ensuring safe and successful CAS [3, 4, 10, 12, 13].

Endovascular robotic work in the arch and great vessels region has demonstrated significant reduction in the number of wall hits and reduction in high intensity transient signals (HITS) recorded on trans cranial doppler (TCD). Our research group studied 44 manoeuvres in 11 patients undergoing thoracic endovascular aortic repair (TEVAR) [8]. The study compared manual versus robotic placement of wire and catheter across the arch. There was significantly lower number of HITS recorded on TCD while using the robotic manoeuvring. A benchtop high fidelity flow study involving robotic and manual cannulation of carotid vessels performed by 17 experienced clinicians showed significant reduction in catheter wall hits, catheter tip movement as well as cannulation time in favour of robotic catheterization [5]. Another pre-clinical study showed significant reduction in the peak and mean contact forces exerted by robotic catheter compared with conventional catheter when cannulating arch vessels [9] The current study of robotic CAS demonstrated high technical and clinical success despite presence of challenging anatomical factors including; aortic arch grade 3 in 61%, arch angle <90° in 77% of patients and proximal TI > 150 in 46% and distal TI > 150 in 23% as well as atheroma grade 4 in 23%.

### Study Limitations

The main limitation is a small and non-comparative series. However, this is the first study we are aware of that assessed objectively the performance and outcome of robot-assisted CAS.

## Conclusion

Endovascular robotic carotid artery stenting is feasible and safe even in challenging arch and carotid artery anatomy. The role of robotics and remote intervention should be appraised in future trials to support technological developments in this field.
